# “Connectedness” between people with intellectual disabilities and challenging behaviour and support staff: Perceptions of psychologists and support staff

**DOI:** 10.1177/17446295211056820

**Published:** 2022-01-27

**Authors:** T Tournier, AHC Hendriks, A Jahoda, RP Hastings, PJCM Embregts

**Affiliations:** Tranzo, Tilburg School of Social and Behavioral Sciences, 120694 Tilburg University, The Netherlands; ASVZ, Sliedrecht, The Netherlands; School of Pedagogical and Educational Science, Faculty of Social Sciences, 159214 Radboud University Nijmegen, The Netherlands; Institute of Health and Wellbeing, 98453 University of Glasgow, Glasgow, UK; Centre for Educational Development, Appraisal and Research, University of Warwick, Coventry, UK; Centre for Developmental Psychiatry and Psychology, Department of Psychiatry, School of Clinical Sciences at Monash Health, Monash University, Melbourne, VIC, Australia; Tranzo, Tilburg School of Social and Behavioral Sciences, 7899 Tilburg University, The Netherlands

**Keywords:** challenging behaviour, person-centred approach, relationship

## Abstract

The tacit practical knowledge of psychologists and support staff to foster a real connection between support staff and people with intellectual disabilities and challenging behaviour was explored. Therefore, six dyads comprising individuals with intellectual disabilities and challenging behaviour and their support staff were video recorded during joint engagement in an activity. To tap into the implicit knowledge of professionals about when staff have ‘a real connection’ with a person with an intellectual disability, 10 psychologists and 10 support staff were asked to pinpoint these moments in the pre-recorded video compilation. They also shared their interpretations about what they considered to be a real connection. The results displayed that participants designated real connections as occurring when they noticed concrete interactions taking place. Based on thematic analysis of the data, four themes were identified that encapsulated what professionals deemed to be a real connection. In conclusion: joint engagement in an activity appears to be a context that fosters opportunities for real connection. Furthermore, support staff should adopt a sensitive attitude and create a safe atmosphere, to establish real connections.

## Introduction

Social relations are essential to health and well-being (Helliwell and Putnam, 2004), and have been linked to both an increased quality of life and a decreased likelihood of experiencing depressive symptoms amongst people with intellectual disabilities (Lunsky and Benson, 2001). Conversely, feelings of loneliness and a lack of social ties have been associated with an increased risk of being diagnosed with a mental illness (Scott and Havercamp, 2014). Moreover, social relations provide people with intellectual disabilities with a sense of satisfaction and enhance their self-esteem (Liang et al., 2001). The significance of social relations is also apparent in the case of people with intellectual disabilities and challenging behaviour. For example, people with intellectual disabilities themselves have reported that positive relationships with peers and family can mitigate against challenging behaviour, insofar as these relationships induce a feeling of safety, belonging and being liked, which, in turn, can generate a sense of contentment, calmness, and security (Clarke et al., 2019).

When people with intellectual disabilities and challenging behaviour are living in service facilities, the social relationships that they have with support staff constitute a large part of their network (Van Asselt-Goverts et al., 2015). Indeed, relationships with support staff are highly valued by people with intellectual disabilities and challenging behaviour. Moreover, support staff have been found to play a significant and meaningful role in their social network, in terms of providing both instrumental support (e.g., supporting them to prepare meals) and emotional support (e.g., listening to their problems; Griffith et al., 2013). People with intellectual disabilities and challenging behaviour characterise such positive relationships with support staff as being trusting, and as having someone who can provide them with clear advice, guidance, support in solving problems, and have fun with them (Clarke et al., 2019). The attributes of support staff that are deemed to facilitate positive relationships by people with intellectual disabilities and challenging behaviour include: having time for them, being competent, being genuinely interested in their lives, and adopting a calm and consistent approach (e.g., Clarke et al., 2019; Van Den Bogaard et al., 2019). Engaging in a positive relationship with support staff can increase their confidence, and, in turn, help them to achieve their goals (Ruef and Turnbull, 2002).

Person-centred approaches, such as Positive Behavioural Support (Carr et al., 2002; Gore et al., 2013), Active Support (Mansell and Beadle-Brown, 2012) and Triple-C (Tournier et al., 2020), emphasise the importance of the relationship between people with intellectual disabilities (and challenging behaviour) and support staff. A ‘real connection’ is an integral part of such a positive relationship, in that it enables support staff to gain insight into, and respond to, the needs of people with intellectual disabilities (Hermsen et al., 2014; Reinders, 2010). To gain insight into the underlying process of how to establish a connection, some researchers have analysed the actions of support staff. For example, Antonsson et al. (2013) focused on successful interactions between support staff and 11 people with various levels of intellectual disabilities (some of whom also displayed challenging behaviour), all with communication difficulties. Their results showed that support staff tailored their language to the individual, ensured that their communication was directly relevant to the activity they were engaged in, as well as using signs and body language to facilitate their understanding. Other studies have also reported that support staff adjust their communication to suit the needs of people with intellectual disabilities, who have limited communication skills. For example, Johnson et al. (2012) adopted a grounded theory approach to analysing data generated via observations and interviews with six people who had severe intellectual disabilities and limited communication skills, and their staff and family members. Adjusting how one communicates was framed as being part of how someone connects with people with intellectual disabilities and limited communication skills.

Both support staff and psychologists have a significant role in implementing and applying person-centred approaches that emphasise the importance of the relationship between people with intellectual disabilities and challenging behaviour and support staff (Stenfert Kroese and Smith, 2018). However, from examining the literature, there has been no research investigating the tacit knowledge of psychologists and support staff about what they consider to be a real connection with a person who has intellectual disabilities, despite the importance of this knowledge in person-centred approaches. Knowledge that ‘is acquired through experience and of which we are unaware’ (Burford and Jahoda, 2012) that guide people’s attunement to others in everyday interactions. Hence it this tacit knowledge was explored by examining: (1) when psychologists and support staff considered moments of real connection as having occurred, and (2) what psychologists and support staff considered to be real connections.

## Method

### Participants

The first step of the research involved six dyads comprising people with various levels of intellectual disabilities and challenging behaviour and their support staff being video recorded in five different group homes based on a residential site of a Dutch service provider. All support staff were male, and their mean age was 34.8 years (range 28–42). Their average work experience in supporting people with intellectual disabilities was 9.2 years (range 8–11). Two support staff attended secondary vocational education, four completed higher professional education. All the people in the videos with intellectual disabilities and challenging behaviour were male, and had varying levels of intellectual disabilities based on the results of the Wechsler Adult Intelligence Scale (Wechsler, 2012; severe = 1, moderate = 2, mild = 1) and, in the case of one participant, the Vineland-3 Scale (Communication: 2; 5 years, Daily Living Skills: 4; 1 year, and Socialization: 1; 5 years; Sparrow et al., 2016). The person with a mild intellectual disability was video recorded twice with two different support staff. The individuals with intellectual disabilities had lived, on average, in the residential facility for 16.6 years (range 6–30), and within their current group home for 5.3 years (range 0.9–9). Four of these individuals were officially diagnosed with autism, one of them also had Attention Deficit Hyperactivity Disorder. Another individual had posttraumatic stress disorder, type I and II. To give a general picture of the challenging behaviour displayed by the participants with intellectual disabilities over the last 2 months, the Dutch Behaviour Problems Inventory-01 (BPI-01; Dumont et al., 2014; Rojahn, et al., 2012) was used. The BPI-01 contains three sub-scales and items are rated on a frequency scale (0 = never to 4 = hourly), and a severity scale (0 = no problem to 3 = severe problem). The participants’ scores for each scale on the BPI-01 are presented in Table 1.

**Table 1.** Mean frequency and severity scores on the BPI-01 for individuals with intellectual disabilities.

		Participant 1 (*M)*	Participant 2 (*M)*	Participant 3 (*M)*	Participant 4 (*M)*	Participant 5 (*M)*
Self-injurious behaviour	Frequency	2.00	1.00	2.25	1.60	2.00
	Severity	2.00	1.00	2.25	2.20	2.00
Stereotyped behaviour	Frequency	2.62	3.67	2.57	1.69	2.00
	Severity	1.77	1.33	2.07	1.38	1.20
Aggressive/Destructive Behaviour	Frequency	2.00	3.00	2.50	1.20	0.00
	Severity	2.00	2.00	2.88	1.20	0.00

In the second step of the research, 10 support staff and 10 psychologists who work with people with intellectual disabilities and challenging behaviour were invited to individually review the video compilation. All these professionals used the Triple-C approach in their work, and, were trained in the Triple-C vision and method. This is a person-centred approach from the Netherlands that is used to support people with intellectual disabilities and challenging behaviour. The approach has a strong focus on relationships between people with intellectual disabilities and support staff. A significant element of the Triple-C approach is for support staff to have an unconditional supportive relationship with the individual with intellectual disability. To do so, support staff need to be able to create a safe and secure (social) environment in which they can attempt to truly connect with the individual with intellectual disability. A more detailed description of the approach can be found Tournier et al. (2020). Given their background training and experience, we expected these staff to have tacit knowledge about ‘connections’ in the interactions between staff and people with intellectual disabilities.

The average age of the support staff was 32.5 years (range 28–44), and eight of them were male. The average duration of their work experience caring for people with intellectual disabilities was 11.5 years (range 3–20). Three participants completed high school, five participants attended secondary vocational education, and two of them attended higher professional education. All but one of the psychologists were female. The average length of time all 10 psychologists had worked in the field of intellectual disability care was 21.2 years (range 7–33). All of them had higher professional education and training in a range of academic disciplines, five of them also had postgraduate degrees in healthcare psychology.

### Procedures

Ethical approval was obtained for the study from the Ethics Committee of [NAME] University (EC-2015.29). The two founders of Triple-C, two skilled Triple-C psychologists and two Triple-C managers were consulted to select both the support staff to record and the support staff and psychologists who would review the video compilation. Support staff that were to be recorded were asked if one of the residents in their group home would like to be recorded together with them while carrying out an activity. The support staff then invited the resident and contacted the resident’s legal guardian to ask for their consent. All individuals gave their informed consent to be video recorded.

#### Video recording

The founders of Triple-C and the recorded support staff were consulted when a connection between people with intellectual disabilities and support staff was likely to occur. Based on their advice, video recordings were made of them during joint engagement in an activity: shopping for groceries, setting the table for dinner, having breakfast, replacing the rubbish bag, and serving coffee to roommates.

The researcher recorded the six dyads on three separate occasions. Where possible, the first 2 minutes of the third video of each dyad was used to produce the compilation video. However, the sections of video had to be of sufficient quality to allow both the support worker and the person with an intellectual disability had to be heard clearly and be continuously visible. When this was not the case in the first 2 minutes of film, then the selection started from the moment the video met the quality standards as described above for a 2 minute period. A compilation of video extracts was selected in order to provide a possible variety of forms of connectedness. In total, the video compilation was 13.37 min long. To capture typical interactions between support staff and people with intellectual disabilities and challenging behaviour, support staff were asked to support people with intellectual disabilities in the same way that they normally would when carrying out an activity together.

#### The Burford review procedure

A video review method was used known as the Burford Review Process (BRP; Burford, 1993; Burford and Jahoda, 2012). This method aims to gain insight into individuals’ intuitive judgements about human action that they witness in video material, that is, what they are drawn to in the observed action. Within this approach, the researcher’s task is to allow participants to make these judgements, without constraining them via the use of pre-determined codes or categories. All participants need to respond to the same video in order to be able to answer the research questions.

The video review process was completed virtually, using Microsoft Teams. As the relationship between the researcher and the reviewer is considered to be one of the essential elements in the BRP (Burford and Jahoda, 2012), the researcher attempted to create a relaxed atmosphere so the participant could settle down, become familiar with the situation, and thus feel at ease to share their thoughts. Reviewers were assured that they were neither under evaluation nor being judged on how well they ‘performed’ or ‘saw’ things, and that they were considered to be expert informants.

The reviewing sessions lasted between 42 to 105 min (*M* = 61.2 min). First, the researcher explained the aim of the study and the procedure, and then the participant was given the opportunity to ask any questions. The review process began with the question: “Could you please say ‘Yes’ when you think the person with an intellectual disability and challenging behaviour and the support worker are ‘connected’?” Prior to the actual data collection taking place, the procedure was tested by showing a small sample of video to the participant, to ensure that the procedure was clear. Then, the official video compilation was shown to the participant and the marked moments were recorded. Finally, the researcher went through the videotape with the participant a second time, looking at each of the pinpointed moments, and asking the participant to explain why they highlighted these moments. The researcher did not engage in any in-depth discussion during this section, but rather asked clarifying questions (Burford et al., 2003). In the end, the participant was given the opportunity to make general comments about the video. The participants’ views were audio recorded and transcribed verbatim.

## Analysis

The analysis was executed in three steps. First, the marked moments for each participant (i.e., selected seconds) were entered into Excel and transferred to SPSS. Then, the average number of identified moments for each participant were calculated.

Second, moments of strong agreement were selected based on the results in step 1. Next, a detailed description was provided of several of these selected situations. No interpretations were made at this stage as to why participants considered these selected moments to be indicative of real connection.

Third, participants’ comments about the selected moments were subjected to thematic analysis (Braun and Clarke, 2006), in order to identify, analyse and report patterns in the data. The data from the psychologists and support staff were individually analysed by two researchers, using an inductive approach. Atlas.ti. software (Friese, 2019) was used to help conduct this analysis. Then, themes and categories were extracted for each subgroup from the codes by the two researchers, based on valid inference and interpretation. The results were then discussed with the whole research team.

## Results

The first research question sought to examine *when* professionals considered a real connection between support staff and people with intellectual disabilities and challenging behaviour to have taken place, by tapping into their implicit knowledge on connectedness. The whole video contained 817 segments that were each a second long. The results showed that, on average, the 20 participants each marked 41 (range 12–93) moments (i.e., seconds) of real connection between people with intellectual disabilities and support staff. For each second of video, on average, one participant marked a moment (*SD =* 1.34). A one second interval marked by two participants was deemed to be statistically significantly different to the expected frequency based on an even distribution of selected moments, *t*(816) = −21.25, *p* < .001. In Table 2, the number of participants that marked the same second as a moment of real connection upward of two participants for each second are displayed. These moments were considered as indicating agreement between participants.

**Table 2.** Frequency of the moments of agreement by the participants.

Number of participants marking the same second	Frequency of the occurrence
2	123
3	53
4	18
5	9
6	8
7	2
8	2
9	1

To answer the second research question, *what* do professionals consider to be a real connection between support staff and people with intellectual disabilities and challenging behaviour, two analyses were carried out. The first was done to gain additional insight into the context in which participants had considered a connection to have taken place. The second analysis was conducted on the interpretations that participants gave about what they considered to be a real connection. With respect to the first part of this analysis, detailed descriptions were made of several examples of moments that were selected by five or more participants (Table 3). This selection was made due to the fact that a considerable number of the participants had marked these particular moments as being indicative of real connection.

**Table 3.** Examples of agreed moments of connection.

Number of participants who marked the same second	General context	Examples of specific description of the context
5	Pouring coffee for roommates	The support worker sits on the couch while the person with an intellectual disability is pouring coffee in a cup. The support worker asks, “Shall I put some milk in it, or will you do that?”
	Picking a piece of fruit to take to work	The support worker and the person with an intellectual disability are both standing by the fruit bowl, while the person with an intellectual disability is grabbing a banana. Both look at what the person with an intellectual disability is doing. Then, the person with an intellectual disability looks at the support worker and says, "Is this healthy too?", while pointing at an orange. The support worker replies with "Vitamins are your friends boy, aren't they?" The person with an intellectual disability says "Yes"
6	Sitting at the breakfast table	The support worker holds the jar of peanut butter and the tub of cream cheese in front of the person with an intellectual disability. Both men are looking at the products. The person with an intellectual disability looks at the support worker and points to the cream cheese. The support worker says "Cream cheese… take your knife"
	Changing the rubbish bag	The support worker walks up to the person with an intellectual disability (who is opening a new rubbish bag), clenches his fist and asks, "Would you like to try out your new tools?" He stands next to the person with an intellectual disability who is looking at him and says, "Yes, I would like to try out my new tools that I bought on Saturday"
7	Sitting at the breakfast table	The person with an intellectual disability slides their arm across the table towards the hand of the support worker. The support worker puts his hand on the person with an intellectual disability while they make eye contact. Then, the support worker releases the person with an intellectual disability’s hand, looks towards his coffee cup while asking, "What have you done yesterday?"
8	Doing groceries together	The support worker points towards a shelf while saying “That one?” The person with an intellectual disability looks where the support worker is pointing and picks a particular product
9	Setting the table	The support worker stands at the counter and hands two glasses to the person with an intellectual disability while saying “Look, you can put these by the plates”. While saying this, he holds the glasses a bit longer, and the person with an intellectual disability, who is holding the glasses as well, looks him in the eyes

The results in Table 3 depict concrete interactions between people with intellectual disabilities and challenging behaviour and support staff, including having brief conversations, making eye contact, and engaging in other forms of physical contact. Another notable result is that in all these situations support staff play an active and prominent role, for example, by giving instructions, making gestures or handing over tableware to set the table. In these scenarios, the people with intellectual disabilities often appear to be highly responsive towards the actions of support staff.

The second part of the analysis, which sought to understand *what* professionals consider to be real connections between support staff and people with intellectual disabilities and challenging behaviour, involved conducting a thematic analysis. The thematic analysis encapsulated four themes in participants’ explanations of what constituted a real connection: (1) the way in which connections between support staff and people with intellectual disabilities become visible; (2) support staff creating a safe atmosphere; (3) support staff attuning to the needs of people with intellectual disabilities in a sensitive way; and (4) people with intellectual disabilities attempting to connect with their support staff. In the description of these themes, the explanations of the reviewing psychologists and support staff were drawn upon.

### Theme 1: The way in which connections between support staff and people with intellectual disabilities become visible

The first theme describes participants’ explanations of what they deem to be real connections between support staff and people with intellectual disabilities and challenging behaviour. The theme was built upon two subthemes: *joint engagement in a meaningful activity* and *visible connection.*

#### Joint engagement in a meaningful activity

Both psychologists and support staff routinely talked about joint engagement in meaningful activity being an indicator of a real connection. They considered the joint share in an activity as the sign of a connection.

Now you see the connection. I actually think it is because they are now working on something together again with that sandwich. I can see immediately that the client calms down. [Psychologist 6]

Support staff often referred to a real connection as an ‘invisible line’ between support staff and people with intellectual disabilities and challenging behaviour. That is, even in the absence of explicit forms of contact, both people on the video knew exactly what had to be done in the activity, while the person with an intellectual disability was capable of executing the activity without the need for too much support.

Now you can see that the support worker is not focused on the client but knows exactly what the client is doing. I think that may actually be the most special connection; that you are not involved with each other, that you do not see each other, but that you know exactly what you can expect from each other at that moment. So, even though there is no real contact, there is a connection. [Support worker 1]

Participants sensed that people with intellectual disabilities and challenging behaviour and support staff paid attention to each other during the joint activity; they observed that the people in the video were following each other’s actions when they worked together. During joint engagement in meaningful activities, the participants also believed that a connection took place when both people with intellectual disabilities and support staff acted in the ‘here and now’. That is to say, they were not distracted in any way; rather, their focus was explicitly on the other while carrying out the activity together.

#### Visible connection

Participants frequently acknowledged explicit forms of connection while support staff and people with intellectual disabilities engaged in joint activities. Eye contact and both verbal and physical forms of contact were all indicators of a real connection. Different types of verbal contact were highlighted as indicating a connection. For example, when a support worker noticed that the person with an intellectual disability was tense, they tried to understand what was causing this tension by asking the person with an intellectual disability a question:

*Yes, that piece is beautiful. “What are you looking at?” So, he* [support worker] *noticed again, he sees the tension, “what are you looking at?” …, even when he sees* [name person with intellectual disability] *is actually with his thoughts somewhere else, then I* [support worker] *will try and put myself into his thoughts. So, that is what I think again, he is constantly looking for reciprocity. So, I like that very much*. [Psychologist 1]

Verbal contact was also used when support staff sensed that people with intellectual disabilities and challenging behaviour were more at ease, and sought to connect to them via engaging in brief conversations. The participants considered this to be a means through which to show genuine interest in people with intellectual disabilities, and to have an equal conversation.

In some instances, this verbal contact was combined with physical contact. For example, in one scenario a support worker put their hand on the shoulder of the person with an intellectual disability while giving him instructions. Participants indicated such forms of physical connection can have different effects on people with intellectual disabilities, such as providing comfort and reassurance.

Finally, eye contact between support staff and people with intellectual disabilities was also seen as a sign of connection by both groups of participants. Different attributions were given to this type of contact, such as the support worker checking if there was still a connection, reassuring the person with an intellectual disability during an activity, and letting them know that the support worker was still there for them.

Yes, that was exactly that moment of eye contact, where the support worker nods, “I understand you”. I do not know exactly what [name person with intellectual disability] was talking about, but the support worker lets the person know “I hear you and I understand you”. [Support worker 5]

### Theme 2: Support staff creating a safe atmosphere

In addition to the interpretations of moments of connectedness, the participants also noted that the recorded support staff created a safe atmosphere for people with intellectual disabilities and challenging behaviour. According to the comments of the participants, this atmosphere was considered significant for being able to connect with people with intellectual disabilities. This theme was built upon three subthemes: *support staff creating a familiar and reassuring feeling*; *support staff displaying an approachable attitude*; and *support staff being confirmative and complimentary*.

#### Support staff creating a familiar and reassuring feeling

The psychologists talked about support staff evoking feelings of familiarity and reassurance in order to connect with people with intellectual disabilities. By creating a familiar environment, people with intellectual disabilities were able to actively join in the mutual activity, which was considered to be indicative of a connection. In addition, psychologists reported that when support staff noticed that people with intellectual disabilities were unsure about what they were supposed to be doing in an activity, or were feeling stressed, they displayed a reassuring attitude that helped to maintain the connection.

He [support worker] keeps calling his name, you know, and uh ... Also speaking in a very calm tone. He [support worker] is really doing it together. [Name person with intellectual disability] actually drops out from engaging in the activity 20 times in this video, but he brings him back in 20 times with the same tranquillity and I think that is very reassuring for the client. [Psychologist 1]

#### Support staff displaying an approachable attitude

Psychologists and support staff also regarded the attitudes of the support staff as being significant for establishing connections with people with intellectual disabilities. When support staff adopted a respectful, kind, and calm attitude during the execution of a joint meaningful activity, the participants noticed a connection. Participants indicated that this is because people with intellectual disabilities are more willing to connect with support staff when they feel at ease with their carers’ approachable attitude.

I think it is just above all his calmness and the sense of equality that he radiates ... I think that is his strength… [Psychologist 1]

#### Support staff being confirmative and complimentary

Both groups of participants mentioned that recorded support staff where confirmative and gave compliments while carrying out meaningful activities together. This behaviour of recorded support staff was considered to contribute to create a safe atmosphere in which a real connection could occur.

… [support worker] considers it for a moment, and also approving of euh ... a nod with his head, like things are going well you know. A very small compliment that he [support worker] gives him very often, so that he [person with intellectual disability] really realises that it is going well. You are doing well, a kind of confirmation of it is going well. [Support worker 6].

### Theme 3: Support staff attuning to the needs of people with intellectual disabilities in a sensitive way

The third theme pertains to the sensitive attitude displayed by support staff. Both groups of participants considered that the sensitivity of staff played a significant role in terms of fostering a connection. This sensitive attitude was understood as showing genuine interest in people with intellectual disabilities, trying to place themselves in their mind and attempting to ascertain what they felt and needed. This theme was built upon two subthemes: *support staff adjusting their proximity and pace to establish a connection*; and *support staff adjusting their actions to foster a connection*.

#### Support staff adjusting their proximity and pace to establish a connection

Participants described the support staff as being sensitive when attuning their proximity to the needs of people with intellectual disabilities. That is, when support staff noticed that they had to be close to people with intellectual disabilities in order to be connected while executing a joint activity, or if they needed to give them more space. For example, one support worker noticed that the person with an intellectual disability was stressed and reached out to make a physical connection with them. In order to maintain this connection, the support worker made physical contact by placing their hand on the hand of the person with an intellectual disability:

*Yes, what I like, is that he* [support worker] *sees that hand coming towards his hand again, …, he* [support worker] *literally puts his hand on his hand for a moment, then lets it go again, you know, so he plays with the connection and really looks at him* [person with intellectual disability] *a little longer. Then he asks another question. So, he felt that tension well again.* [Psychologist 1]

Conversely, another support worker opted to take a step back when he sensed that the person with an intellectual disability could manage the activity independently.

There is a clear goal, but what I like most about this situation is that the client gets around three or 4 m of space from the support worker at some point, and I think that might be the best connection there is. Like, okay, I see you, I am here for you, I will help you, but go do it by yourself. I think that is the most beautiful kind of connection in this situation. [Support worker 1]

Participants also commented on how support staff slowed down their actions to develop a connection with people with intellectual disabilities. By taking their time and letting people with intellectual disabilities respond in their own time, support staff were able to truly connect to the person they were supporting.

#### Support staff adjusting their actions to foster a connection

Participants expressed that support staff used multiple actions to either become or maintain connected. Although the actions described in this part of the results section were mentioned independently, combinations of these actions were also mentioned. Examples of such actions were giving instructions, using gestures (pointing towards something), demonstrating (a part of) the activity themselves, or using objects (a glass or a plate) to clearly illustrate what needed to be done in the activity. Participants also observed that support staff used small sounds (making noises with cutlery) to maintain the attention of people with intellectual disabilities and thereby stay connected. To reduce the use of verbal language, in some cases support staff made their intentions clear by explicitly looking at something, so the person with an intellectual disability would also look at it and they would have a moment of shared attention:

Yes, I actually just think the moment he passes it to him, and you also see where you normally see people making eye contact, here you see [name support worker] is now not looking on purpose, [name person with an intellectual disability] also does not look, in my opinion, and actually they both have the same position, so [name support worker] looks down and [name client] also looks down. While, yes, [name support worker] is not really busy with anything. He just looks at something, or down, to make [name client] also look down. So, I like it, how he does that trick. [Support worker 11]

Other actions that were described as being used to either become connected or maintain connection were when support staff captioned their own actions (i.e., saying out loud what they were doing), turning and leaning with their body explicitly towards the person with an intellectual disability, or, alternatively, used humour to establish a connection.

### Theme 4: People with intellectual disabilities attempting to connect with their support staff

The final theme is based on several statements from psychologists and support staff, who focused on the perspective of people with intellectual disabilities and challenging behaviour in establishing connections. Despite the fact that this perspective was rarely mentioned by the participants, the theme is nevertheless considered relevant. Two subthemes were distinguished: *participating in the activities of support staff* and *engaging in actions to become connected*.

#### Participating in the activities of support staff

Psychologists and support staff considered a connection to have taken place when people with intellectual disabilities participated actively in an activity together with their support staff. That is, when people with intellectual disabilities effectively responded to what support staff asked of them.

We have to buy that together and then you can get it. So, that you really do it together…, I point it out, you take it and put it in the shopping cart. [Psychologist 10]

#### Engaging in actions to become connected

Both groups of participants noted that, in some instances, people with intellectual disabilities actively asked for reassurance, such as by making eye contact or looking at their support worker. In some situations, people with intellectual disabilities even asked support staff questions.

And I also think it is funny, he [person with intellectual disability] asks a question and [name support worker] responds to that, so that is also ... he [support worker] just lets him ask his question and he [support worker] gives him an answer. So, that [name person with intellectual disability] can also respond again, so a small dialogue occurs. In that sense, they do have contact. [Support worker 3]

Finally, several of the psychologists discerned that people with intellectual disabilities turned their body explicitly towards their support worker, in a concerted effort to connect with them.

## Discussion

This study aimed to gain insight into what psychologists and support workers perceive as ‘a real connection’ between people with intellectual disabilities and challenging behaviour and support staff, by examining their tacit knowledge. The study used the novel Burford method (1993) to capture psychologists and support workers’ intuitive judgements about video interactions that they reviewed, without being constrained by the use of pre-determined codes or categories. The findings suggest that this may be a way of obtaining new understanding which challenges preconceived ideas in the field.

The first research question examined *when* psychologists and support staff considered moments of real connection to have occurred. The results indicate that there were many moments of agreement about when participants considered that a real connection had taken place between support staff and people with intellectual disabilities and challenging behaviour. Indeed, in one case, nine people selected the exact same second as indicating a moment of connectedness. The second research question, *what* psychologists and support staff considered to be a real connection, pertained to situations in which a concrete interaction was occurring (e.g., verbal or physical contact). In most of these situations, support staff played a prominent role and people with intellectual disabilities often appeared to be responsive towards the actions of support staff. Furthermore, the thematic analysis of the interpretations of professionals about *what* constituted a real connection showed that, a real connection could occur when people with intellectual disabilities and support staff were engaged in a joint meaningful activity, and, when there was a visible connection between them (verbal, eye or physical contact). In addition, the participants indicated that to be able to establish a connection with each other, support staff had to create a safe atmosphere that produced a familiar and reassuring feeling for people with intellectual disabilities. Besides this, support staff should also display an approachable attitude and be confirmative and complimentary towards people with intellectual disabilities. Finally, the results indicate that it was necessary for support staff to adopt a sensitive attitude in order to foster a connection between people with intellectual disabilities. By attuning to their needs, in terms of proximity, pace and other types of actions (e.g., the use of gestures, demonstrating the activity, etc.), the participants deemed that support staff were able to connect to people with intellectual disabilities and challenging behaviour. It is also noteworthy that both groups of participants primarily described connectedness from the perspective of support staff, with the perspective of people with intellectual disabilities and challenging behaviour rarely being mentioned. In those rare instances in which the participants did note that people with intellectual disabilities were attempting to connect, it was because they were either participating in the activity together with the support worker or explicitly attempting to connect, by, for example, asking a question or making eye contact.

These results suggest that joint engagement in an activity is considered to be a significant context for establishing connections. Active participation in daily life via engaging in meaningful activities is also a core element of multiple approaches (e.g., Positive Behaviour Support, Active support, Triple-C), which seek to support people with intellectual disabilities experience a life as close as possible to an “ordinary life” (King’s Fund Centre, 1980). In the case of Triple-C, joint engagement in a meaningful activity is also one of the core assumptions regarding how to build a positive relationship between people with intellectual disabilities and challenging behaviour and support staff; however, this assumption needs to be underpinned by scientific evidence (Tournier et al., 2020).

Furthermore, the need for support staff to display sensitive attitudes and create a safe atmosphere was also considered to be integral to establishing a connection. Based on the interpretations of the participants, support staff were able to sufficiently meet the needs of people with intellectual disabilities and challenging behaviour, which, in turn, led to moments of real connection. In addition, the participants expressed that support staff attuned themselves to the needs of people with intellectual disabilities as a way of generating feelings of reassurance, comfort, mutuality and genuine interest. This finding is in accordance with previous research, which similarly highlighted the importance of attunement (Reuzel et al., 2017) and showed that evoking such feelings confirms the humanity of the person who is dependent upon care (Antonsson et al., 2013; Hermsen et al., 2014). When viewed in the context of ours results, this could explain why the participants considered these moments to be indicative of a real connection. Besides, our results are also in line with a recent concept mapping study by Nijs et al. (2019), which indicated that support staff should attune to the needs of people with intellectual disabilities and challenging behaviour in order to strengthen the connection. According to Simons et al., (2020), support staff should assess the support needs of people with intellectual disabilities and challenging behaviour across a range of dimensions (i.e. cognitive functioning, adaptive behaviour, participation, health, and context), and subsequently tailor their support to their clients’ needs with respect to these dimensions. Based on their systematic review, Simons et al., (2020) argued that support staff should have knowledge about people with intellectual disabilities, their own psychological resources, their own causal explanations for understanding the challenging behaviour, along with adopting an optimistic, friendly and understanding attitude, and reflecting upon how they cope with their own emotions during their work (Simons et al., 2020).

The feelings that the participants highlighted as being indicative of connections between people with intellectual disabilities and challenging behaviour and support staff share similarities with those cited by people with intellectual disabilities and challenging behaviour as being important for developing a positive relationship. Prior research has shown that people with intellectual disabilities and challenging behaviour view trust, having genuine interest, and displaying a calm approach to all be important qualities for support staff to have in terms of building a positive relationship (e.g., Clarke et al., 2019; Griffith et al., 2013; Van Den Bogaard et al., 2019). However, further research is needed to learn how people with intellectual disabilities and challenging behaviour themselves experience connections with support staff. This is underpinned by the results of the current study, which showed that both psychologists and support staff primarily focused on the connection from the perspective of support staff. Including the perspectives of people with intellectual disabilities and challenging behaviour is thus necessary, because such connections are built on two-way interactions, and, hence, the experiences of both parties are of equal importance (Antonsson et al., 2013).

However, the findings of this study must be considered in light of some limitations. First, all the participants were trained in, and experienced users of, the Triple-C approach. This approach has a strong vision about how to build relationships between people with intellectual disabilities and challenging behaviour and support staff. A key assumption of Triple-C is that this relationship is predicated on carrying out meaningful activities together. Consequently, this could have implications for why participants considered engagement in meaningful activity to be a means through which people with intellectual disabilities and support staff connected with one another. In light of this, future research should thus seek to include participants with other backgrounds in the care for people with intellectual disabilities and challenging behaviour as well, in order to examine if they also consider these same moments to be indicative of real connections and provide similar interpretations.

Second, the selection of moments of agreement could be considered to be arbitrary, in that the nature of the obtained data made it difficult to compose consistent inclusion criteria. There were several reasons for this difficulty. The moments of agreement were selected for each second, and thus we did not consider the response times of the participants. This decision was made because some participants selected multiple moments of connection that were close to each other. Moreover, it was hard to decide on a strict cut-off point for what would be considered as a moment of agreement. In other words, how many participants need to select the same second in order for it to be considered a real connection?

A final limitation is that, although engaging in a meaningful activity together appears to be a significant context in which to establish connections, this study only included moments in which people with intellectual disabilities and support staff engaged in activities together. Prior to data collection, Triple-C professionals were consulted when connections between support staff and people with intellectual disabilities and challenging behaviour were likely to occur. Based on their advice, situations were recorded in which support staff and people with intellectual disabilities and challenging behaviour carried out activities together. Due to this selection procedure, we cannot be sure if a real connection only occurs in such situations. Furthermore, another effect of only including routinely occurring meaningful activities is that the invisible line referred to by the participants in this study may be familiarity with performing the same activity on numerous occasions. This may explain why both parties in the video already knew what had to be done during the activity. It may also have affected support staffs’ behaviour, in that they may have been less active than normal in the video due to the fact that the person with an intellectual disability already knew what had to be done, and, as such, required less support. Despite these limitations, the present research has nevertheless shed light on both when a real connection has occurred and what precisely constitutes a real connection between people with intellectual disabilities and challenging behaviour and their support staff when engaging in meaningful activity together. Future research could focus on exploring both when a real connection occurs and what constitutes a real connection when people are either less obviously engaged in an activity together.
